# Evaluation of gastrointestinal injury in blunt abdominal trauma "FAST is not reliable": the role of repeated ultrasonography

**DOI:** 10.1186/1749-7922-7-2

**Published:** 2012-01-20

**Authors:** Afshin Mohammadi, Mohammad Ghasemi-rad

**Affiliations:** 1 Department of Radiology, Urmia University of Medical Sciences, Urmia, West-Azerbaijan, Iran; 2 Student research committee, Urmia University of Medical Sciences, Urmia, Iran

**Keywords:** Ultrasonography, Gastrointestinal, Trauma, FAST

## Abstract

**Background:**

To determine the diagnostic Accuracy of Focused Assessment Sonography for Trauma (FAST) and repeated FAST in the patients with blunt abdominal trauma.

**Methods:**

In this retrospective study we collected the data of all patients from September 2007 to July 2011 with gastrointestinal injury. The intraoperative outcome was compared with FAST technique and the repeated or delayed sonography.

**Results:**

A total number of 1550 patients with blunt abdominal trauma underwent FAST in a period of 4 years in our hospital. Eighty-eight (5.67%) patients were found to have gastrointestinal injury after exploratory laparotomy. Fifty-five (62.5%) patients had isolated gastrointestinal injury and 33 (37.5%) patients had concomitant injury to the other solid organs. In those with isolated gastrointestinal injury, the sensitivity of FAST was 38.5%. Repeated ultrsonography was performed in 34 patients with false negative initial FAST after 12-24 hours. The sensitivity of repeated ultrasonography in negative initial FAST patients in detection of gastrointestinal injury was 85.2% (95% CI, 68.1%, and 94.4%).

**Conclusion:**

Repeated sonography after 12 to 24 hours in patients with negative initial FAST but sustain abdominal symptom can facilitated a diagnosis of GI tract injury and can be as effective method instead of Computed tomography in developing country.

## Background

Trauma is the most common cause of mortality in 1-45 year's age group [1]. Currently ultrasonography (US) is the primary method of screening patients with blunt abdominal trauma (BAT) worldwide [1-3]. Focused Assessment Sonography for Trauma (FAST) has been previously described for the evaluation of blunt abdominal trauma to observe the presence of free fluid in the abdomen or pelvis [4].

Although in some of the previous published literature they believe that it is rare to see false-negative results when screening with US (1%) [5,6]. It seems that screening BAT with FAST will lead to under diagnosis in some abdominal injuries such as; retroperitoneal (pancreatic and adrenal), vascular injuries and diaphragmatic rupture that may have a negative impact on the patients outcome [7].

Due to subtle findings FAST has been reported to be of less value in detection of bowel and mesenteric injuries [8]. Although it is uncommon to develop hollow visceral organ injury after BAT but they are very important to diagnose, because there is no conservative treatment for these types of injuries and all of the patients with such injuries even in unequivocal cases, they need to undergo operative intervention [9]. According to the previous reports the morbidity of gastrointestinal tract injury is mostly related to delays diagnosis [10].

Because of less availability of computed tomography in developing country, the purpose of our study was to determine the role of repeated abdominal US in the patients with negative " FAST "to early diagnose hollow viscous organ injury in patients with BAT. To our best knowledge this is the first report evaluating the role of repeated abdominal sonography to determine and reduce missed gastrointestinal injury by FAST technique.

## Methods

**Figure 1:**
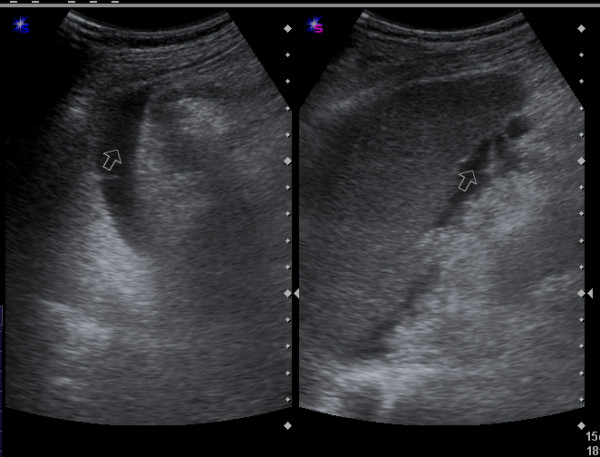
**Longitudinal sonogram show free fluid (arrow) associated with Ileal perforation in pelvic cavity**.

**Figure 2:**
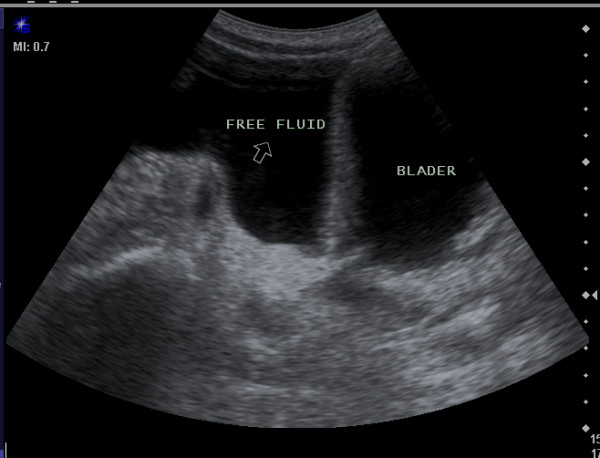
**Ultrasonogram revealed free fluid in the paracolic gutter (right) and perisplenic (left)**.

This retrospective study was started from September 2007 to July 2011. On thousand five hundred and fifty emergency ultrasonography with FAST technique were performed in our University hospital in order to detect free intra-abdominal fluid as an indicator of intra-abdominal organ injury in-patient with BAT (Figure 1, 2).

The outcome of FAST technique and the data regarding type of abdominal injuries were obtained by retrospectively going through patient's operation notes. After retrospectively reviewing the operation record of 1550 BAT patients, 88 were found to have gastrointestinal injury. This study was performed in Imam training University Hospital that serves as the only trauma referral center in our provenance. University review board and ethic committee approved the study.

All the injured patients were referred to our center, maximum one hour after trauma and US examination was performed during first 30 minutes of admission. Examination was performed by one radiologist in the department of radiology at the emergency room. FAST technique was performed by using Sonoline G 40 ultrasound devise (Siemens, Germany) with 3.5-5 MHZ convex transducer. Six areas of the abdomen were examined to detect free fluid; left upper quadrant (LUQ), Morrison pouch, right upper quadrant (RUQ), pelvis, right and left para-colic gutters.

Abdomen and pelvic spiral Computed Tomography (CT) Scan examinations were performed only with IV contrast (Toshiba; X-vision scanner) in 39 patients with BAT and negative FAST after 12-24 hours due to worsening of clinical problem but stable hemodynamic condition.

Spiral CT scans were performed with 10-mm collimation and a table speed of 10 mm/sec. Images were reconstructed at 7-mm intervals. In adults, a total of 120 ml of Iohexol (Omnipaque, 300 mg/50 cc) was administered intravenously at a rate of 3-4 ml/sec. Another experienced radiologist interpreted all of the abdominal CT scans.

The routine protocol in our center is that every patient with suspected abdominal trauma should undergo FAST. Except for those patients that further delaying to intervene to undergo FAST is not possible and the patients need to directly go to the operation room. Those patients with unstable hemodynamics and observable fluid in the peritoneal cavity should immediately undergo laparotomy. Patients with stable hemodynamics and positive sonography will undergo conservative management and close observation.

Those with negative clinical signs and negative FAST are not followed by any other diagnostic methods. But in those patients with negative FAST and constant abdominal pain and stable hemodynamic due to shortage of intravenous contrast material in our center they have to undergo repeated FAST after 12 to 24 hours.

The results of FAST technique were compared with surgical results. Statistical analysis was performed to determine the sensitivity and 95% confidence interval were calculated and used for determining the diagnostic accuracy.

## Results

Out of 1550 patients with BAT a total number of 352 patients (44%) underwent operation. Eighty- eight (5.67%) patients had gastrointestinal injury in exploratory laparotomy (66 (75%) were male and 22 (25%) were female).

The mean age was 28.9 ± 16.5 years (Age range: 3-80 Years). Seventy-one (80.6%) patients had abdominal tenderness during primary physical examination. Forty-seven (53%) patients had stable hemodynamic condition and 41 (46.5%) patients were hypotensive at the time of US examination.

Fifty-five (62.5%) patients had isolated gastrointestinal injury and 33 (37.5%) patients had concomitant injury to the other solid organ such as spleen (n = 14), liver (n = 13), Diaphragm (n = 2), Pancreas (n = 2) and kidney (n = 2).

Emergency US with FAST technique was positive for free fluid in 49 (55.6%) patients (True positive) and was negative (false negative) in 39 (44.3%) patients with gastrointestinal injury.

From 49 patients with true positive FAST, 28 (57.1%) patients had solid organ injury concomitant with bowel injury and 21 (42.8%) patients had isolated gastrointestinal injury. A total of 55 (62.5%) out of 88 patients had isolated bowel injury; FAST exam was positive only in 21 (38.1%) patients (True positive) and was negative in 34 (61.8%) patients. In 34 patients with isolated gastrointestinal injury FAST was negative for free fluid (False negative).

In 39 (44.3%) patients with BAT that the result of emergency US did not show free intra peritoneal fluid in 34 patients, the underwent conservative management and after 12-24 hours serial physical examination showed abdominal tenderness and guarding and worsening of abdominal pain. Upon repeated ultrasonography there was free intra-peritoneal fluid in 29 patients and negative results in 10 patients. All those patients (39 patients) underwent abdominal and pelvic CT, which revealed hollow viscous organ injury in 24 (61.5%) patients. In 15 (38.4%) patients CT examination did not show gastrointestinal injury (false negative) all of which underwent surgical operation because of sustained guarding and unstable hemodynamic condition.

The sensitivity of FAST for detection of gastrointestinal injury in those patients with isolated gastrointestinal injury, the sensitivity was 38.5% (95% CI, 23.2%, and 53.7%).

From 34 patients with negative initial FAST the repeated ultrasonography revealed free fluid in 29 patients and was negative in 5 patients then the sensitivity of repeated ultrasonography in negative initial FAST in detection of gastrointestinal injury was 85.2% (95% CI, 68.1%, and 94.4%).

The sensitivity of CT for the detection of specific sign of gastrointestinal injury such as free air and bowel thickening in the entire study group was 61.5% (95% CI, .44.6%, 76.1%).

The distribution of gastrointestinal injury in these 88 patients is presented in table 1 and distribution of concomitant solid organ injury is presented in table 2.

**Table 1:** table shows the distribution of gastrointestinal injury in trauma

Location	Number	Total
Small bowel		71
Duodenum	7	
Jejunum	36	
Ileum	28	
Large bowel		17
Ascending colon	3	
Sigmoid colon	10	
Transverse colon	4	

**Table 2:** table shows the distribution of concomitant solid organ injury is trauma patients

Location	Number
Spleen	14
Liver	13
Kidney	2
Diaphragm	2
Pancreas	2

## Discussion

Rapid diagnosis and treatment of abdominal injury is an important step to prevent death in BAT patients [1].

Physical examination is frequently unreliable in the setting of acute trauma [11].

Many of the previous reports show that emergency ultrasound is effective in diagnosis of hemo-peritoneum [1,12-14]. Now FAST technique has gained popularity and is been accepted as a diagnostic modality for evaluation of patients with trauma [1,10-15]. Our previous experience showed that sensitivity of FAST in the diagnosis of BAT is 95.4%[1].

MacGahan et al reported free fluid in only three patients with isolated bowel and mesenteric injury in a series of 500 trauma patients [7]. There are several articles pointing that some important abdominal organ injury can be missed by ultrasonography. Dolich et al reported a large number of abdominal injuries (33%), which required operation and were missed in US examination [16].

Shanmuganathan et al showed that 34%(157 patients) of 467 patients with BAT had no free fluid in emergency US [13]. He studied more than 11,000 patients with BAT and concluded that the FAST technique may frequently miss patients with surgically correctable injuries.

Previous reports are indicative of a limited value for FAST in the diagnosis of certain type of injuries such as; diaphragmatic rupture [17], pancreatic [15] and mesenteric injury [18-20].

MacGahan JP et al demonstrated a sensitivity of 44% for diagnosis of isolated gastrointestinal injury by FAST [21]. They also showed that free abdominal fluid was not detected in the majority of patients with isolated bowel and mesenteric injury. Observation, serial physical abdominal examination, Clinical suspicion for bowel and mesenteric injury and CT can all be of help to diagnose intra-abdominal organ injuries.

In our study 39 patients with negative initial US examination and persistent abdominal pain and tenderness underwent repeated ultrasonography after a period of 12-24 hours. Repeated US detected free intra-peritoneal fluid in 29 patients.

Diagnosing gastrointestinal trauma is difficult based on emergency rooms physical examination [19-21] and necessitates using other imaging modality such as CT scan [22,23].

CT has been reported to have a sensitivity ranging from 93-100% in detection of bowel and mesenteric injury. Mirvis et al prospectively detected bowel and mesenteric injury in 17 (100%) patients undergoing laparotomy [22].

Atri et al showed that sensitivity of the three observers in diagnoses of surgically important bowel or mesenteric injury by CT scan ranged from 87%-95% [23]. They concluded that multi-detector CT has high negative predictive value and can accurately show important bowel or mesenteric injuries.

**Figure 3:**
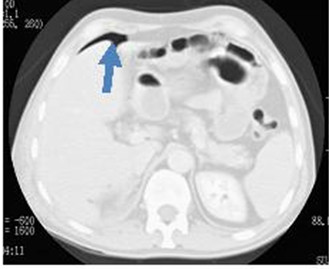
**Abdominal CT scan with lung window shows free air adjacent to liver edge due to colon perforation**.

Levine et al [24] reported that only bowel wall thickening and free air were specific finding in the CT scanning (Figure 3).

And other sign such as, free fluid are nonspecific not reliable to differentiate between bowel and solid organ injuries.

The sensitivity of CT for diagnosis of gastrointestinal trauma in our study is lower compare to other studies [22,23,25], because they used multi-detector CT that is more accurate in diagnosis of GI tract pathology.

McGahan JP et al reported that 49% of the patients with gastrointestinal injury had concomitant injury to other solid organs. The results of our study showed that 38% patients with blunt abdominal trauma had concomitant solid organ injury.

In our study jejunum and ileum were the most common sites of gastrointestinal trauma respectively. The most common solid organ injury concomitant with gastrointestinal trauma was spleen followed by the liver, which were similar to the report by Richards JL et al [18].

The limitations of our study are; single detector CT which can miss some of the intra-abdominal injuries, the retrospective part of the study which we might have missed some of the data in the records, patients with subtle injury such as mild intestinal hematoma may not show clinical symptom and could be missed because they did not underwent repeated abdominal sonography, Inability to calculate the specificity, positive predictive and negative predictive value Since the small injuries could not be seen and consequently are not going to the be operated on.

It is difficult to diagnose gastrointestinal trauma when FAST is performed immediately after admission. As is shown in our report only 38.5% of the patients with free fluid in the abdomen on initial FAST had isolated gastrointestinal trauma. We recommend performing a serial US when CT is not available in-patient suspected of GI trauma and persistent abdominal pain and tenderness, which can reduce the risk of missing major intra-abdominal injuries.

## List of abbreviations

CT: Computed tomography; FAST: Focused Assessment of Sonography for Trauma.

## Competing interests

The authors declare that they have no competing interests.

## Authors' contributions

All the authors in this manuscript have read and approve the final manuscript. AM: Concept, design and the Ultrasonographic studies MG: Manuscript writing and editing and Data analysis.
